# Different effect of testosterone and oestrogen on urinary excretion of metformin *via* regulating OCTs and MATEs expression in the kidney of mice

**DOI:** 10.1111/jcmm.12922

**Published:** 2016-07-29

**Authors:** Rui He, Ligen Ai, Dandan Zhang, Lili Wan, Taishan Zheng, Jun Yin, Huijuan Lu, Junxi Lu, Fengdi Lu, Fang Liu, Weiping Jia

**Affiliations:** ^1^ Shanghai Key Laboratory of DiabetesShanghai Institute for DiabetesShanghai Clinical Medical Centre of DiabetesShanghai Key Clinical Centre of Metabolic DiseasesDepartment of Endocrinology and MetabolismShanghai Jiao‐Tong University Affiliated Sixth People's HospitalShanghaiChina; ^2^ Department of PharmacyShanghai Jiao‐Tong University Affiliated Sixth People's HospitalShanghaiChina

**Keywords:** gonadal hormone, OCT2, MATE1, metformin, kidney

## Abstract

The aim of this study was to investigate the effect of testosterone and oestrogen on regulating organic cation transporters (Octs) and multidrug and toxin extrusions (Mates) expression in the kidney of mice and urinary excretion of metformin. 8 week‐old male *db/db* mice were treated with estradiol (5 mg/kg), testosterone (50 mg/kg) or olive oil with same volume. Metformin (150 mg/kg) was injected in daily for successive 7 days. Plasma, urine and tissue concentrations of metformin were determined by liquid chromatography‐tandem mass spectrometry (LCMS) assay. Western blotting and Real‐time PCR analysis were successively used to evaluate the renal protein and mRNA expression of Octs and MATEs. After treatment, the protein expression of Mate1 and Oct2 in testosterone group was significantly increased than those in control group (both *P* < 0.05). The protein expression of Mate1 and Oct2 in estradiol group was significantly reduced by 29.4% and 43.3%, respectively, compared to those in control group (all *P* < 0.05). These data showed a good agreement with the change in mRNA level (all *P* < 0.05). The plasma metformin concentration (ng/ml) in mice treated with estradiol was significantly higher than control and testosterone group (677.56 ± 72.49 *versus* 293.92 ± 83.27 and 261.46 ± 79.45; *P* < 0.01). Moreover, testosterone increased the metformin urine excretion of mice while estradiol decreasing (both *P* < 0.01). Spearman correlation analysis showed that gonadal hormone was closely associated with Mate1 and Oct2 expression and metformin urine excretion in *db/db* mice (all *P* < 0.05). Testosterone and oestrogen exerted reverse effect on metformin urinary excretion *via* regulating Octs and Mates expression in the kidney of mice.

## Introduction

Diabetes mellitus affects over 25.8 million people (8.3% of the total population) in the United States. A total of 1.9 million new cases aged 20 years or older are diagnosed every year 1. In Chinese people aged >20 years, the prevalence of diabetes was 9.7%, which means 92.4 million adults are suffering from diabetes 2. Metformin is the most frequently used oral drug for treatment of hyperglycaemia in patients with type 2 diabetes both in the United States and in China 3, 4. Recently, metformin has gained more attention for its pleiotropic effects, including anti‐neoplastic and anti‐ageing effects 5, 6. However, studies suggested that metformin therapy is characterized by considerable inter‐individual variability in clinical efficacy 7, 8. Over 30% patients receiving metformin monotherapy were classified as nonresponders in a U.K. follow‐up study during 3 years 8.

Our previous studies suggested the inter‐individual variability in metformin clinical efficacy resulted from the influence of organic cation transporters (Octs) and multidrug and toxin extrusion proteins (Mates) on metformin pharmacokinetics and pharmacodynamics 9, 10. Genetic mutation of OCT2 (808G→T) slowed down the elimination of metformin from kidney and improved the long‐term glucose‐lowering effect in Chinese type 2 diabetic patients 10. Patients carrying SLC47A1 rs2289669 homozygous A/A had remarkably better glucose‐lowering effect after 1‐year oral metformin and pharmacokinetic results of metformin confirmed that carriers of MATE1 homozygous A/A had notably higher time‐dose effects and slower renal elimination of metformin than others 9. Besides, the A allele frequency in MATE1 (SLC47A1 rs2289669) was approximately equal to 50%, much higher than that of OCT2 (808G→T), which meant the effect of polymorphisms in MATEs on metformin disposition and response might be more important than that in OCTs 9, 10. Moreover, our previous study showed there was a gender difference in plasma lactate concentrations in subjects with type 2 diabetes. And gonadal hormones played an important role on regulating plasma lactate levels in diabetes patients treated with metformin. Estradiol up‐regulated but testosterone tent to down‐regulate lactate levels 11. It was revealed that the mRNA levels and protein expression of rOCT2 in the kidney of males were much higher than that of the females. By using Mate1 knockout mice, Toyama *et al*. elucidated the loss of MATE1 was associated with metformin‐induced lactic acidosis 12. Several animal studies had also revealed that gonadal hormones regulated the expression of transporter proteins (Oct2 and Mate1) in rats 13, 14. On the basis of above evidence, we have been suggested that the gonadal hormones could regulate Octs and Mates protein expression in the kidney, alter the elimination of metformin by affecting the process of transport and excretion, and eventually change the long‐term glucose‐lowering effect of metformin. To test this hypothesis, we conducted this study in db/db mice.

## Materials and methods

### Materials

Metformin (97% purity) was obtained from Sino‐American Shanghai Squibb Company (Shanghai, China). Estradiol powder was purchased from National Institutes for Food and Drug Control (Beijing, China). Testosterone propionate injection was purchased from Shanghai General Pharmaceutical co., ltd (Shanghai, China). All other reagents and solvents were of analytical grade and were commercially available.

### Drug administration

Metformin was formulated in PBS (pH 4.5). In these formulations, metformin was soluble to approximately 5 mg/ml. Estradiol was formulated in 0.5% dimethyl sulfoxide (DMSO) and diluted in PBS at a concentration of 50 mg/ml.

### Animals

All experiments were conducted in accordance with the National Institutes of Health regulations of animal care covered in Principles of Laboratory Animal Care (National Institutes of Health, Bethesda, MD, USA) publication 85‐23 and were approved by the Institutional Animal Care and Use Committee. Eight‐week‐old (at the start of the study) male db/db mice (C57BL//KsJ‐lepr^db^/lepr^db^) were purchased from Model Animal Research Centre of Nanjing University (Nanjing, China). Mice were given free access to standard diet and water and kept on a 12:12 hrs light/dark cycle.

### Study designs

The first series of experiments aimed to investigate the effect of testosterone and oestrogen on Octs and Mates protein expression. Mice were divided into three groups (C, T, E groups; *n* = 10) and respectively injected in olive oil, testosterone (50 mg/kg) and estradiol (5 mg/kg) once a day for successive 7 days. On day 8, mice were killed. Plasma and kidney tissue was collected to determinate the gonadal hormone concentration and expression level of Octs and Mates.

The second series of experiment aimed to test the effect of gonadal hormone combined with metformin on Octs and Mates expression and urinary excretion of metformin. As described above, mice were divided into three groups (MC, MT, ME groups; *n* = 10) and treated with metformin (150 mg/kg) by daily intraperitoneal injection for successive 7 days. After the final drug administration, urine samples were collected during the following time intervals: 0–2, 2–4, 4–6, 6–8, 8–12 and 12–24 hrs post metformin. Mice were killed on day 8. Plasma and kidney tissue was collected to determinate the metformin concentration and expression level of Octs and Mates.

### Sample preparation and LCMS analysis

Metformin concentrations in plasma and urine were assayed by a highly specific and sensitive liquid chromatography‐tandem mass spectrometry method 15. The quantification limit was 10 ng/ml for plasma and urine. Both intra‐ and interday coefficients of analysis variation were <10%.

### Fasting blood glucose in mice

Mouse tail veins were transversely sectioned by scissors, and the tail blood was used to determine fasting blood glucose level using a glucometer (Roche, Basel, Switzerland).

### ELISA determination of testosterone and estradiol levels in mouse serum

Before mice were killed, blood was collected from retro‐orbital sinus, centrifuged at 1200 × g for 10 min. at 4°C, and serum was stored at −80°C until assay. ELISA kits were used for detecting serum concentrations of testosterone and estradiol (Mlbio, Shanghai, China) according to the manufacturers’ instructions.

### Western blot analysis

Proteins were separated by 10% SDS‐PAGE and transferred to polyvinylidene difluoride membranes. The membranes were blotted with 5% skimmed milk and subsequently probed overnight at 4°C with primary antibodies specific for Oct1 (Slc22a1, ab66132), Oct2 (Slc22a2, ab179808), Mate1 (Slc47a1, ab105049), Mate2 (Slc47a2, ab174344) and Gapdh (ab8245; Abcam, Cambridge, UK) and then incubated with horseradish peroxidase conjugated goat anti‐rabbit or antimouse secondary antibody. After washing with TBST buffer, the membranes were developed using enhanced chemilu‐minescent reagent and subjected to autoluminography for 1–5 min.

### RNA extraction and quantitative reverse transcriptase PCR

The kidney organs were frozen in liquid nitrogen immediately after dissection and stored at 70°C until analysis. Total RNA was extracted using TRIzol reagent (Invitrogen, Carlsbad, CA, USA). Complementary DNA was subsequently synthesized using a reverse transcription reagent kit (Toyobo, Shanghai, China). The expression level was then evaluated by LightCycler^®^ 96 system (Roche Inc.) using FastStart^™^ PCR Master for Oct (Slc22a1), Oct (Slc22a2), Mate1 (Slc47a1) and Mate (Slc47a2). The Gapdh was used as an internal control.

### Statistical analysis

For statistical analysis, SPSS v. 16.0 (SPSS Inc., Chicago, IL, USA) was used. Figures were created by GraphPad Prism 5.0 (GraphPad Software, LA, USA). Data were expressed as mean ± S.D. for continuous variables. Differences among the groups were analysed using the Kruskal–Wallis test or anova as appropriate for measurement data. Spearman correlation analysis was used to evaluate association between different parameters. All *P*‐values were two‐tailed, and *P* < 0.05 was considered to be statistically significant.

## Results

### The effect of gonadal hormone on Octs and Mates expression

**Figure 1:**
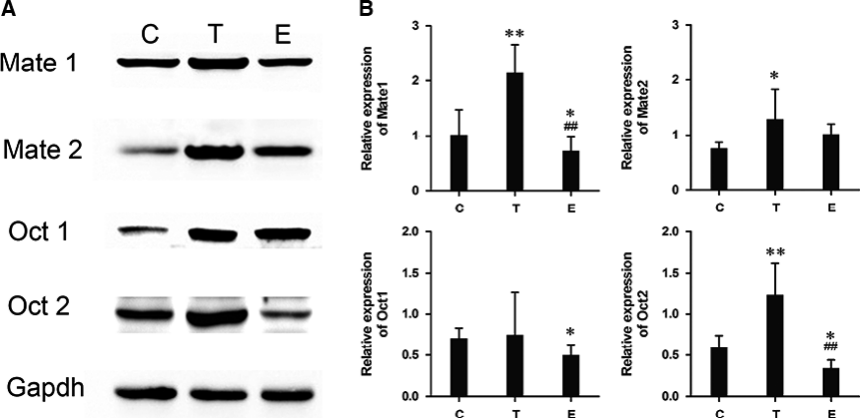
Series 1‐7 days gonadal hormone treatment changed Mate1, Mate2, Oct1 and Oct2 proteins expression in kidney tissue among control, testosterone‐treated and oestrogen‐treated groups by Western blot. (**A**) Representative Western blot; (**B**) the cumulative results of Western blot analysis of Mate1, Mate2, Oct1 and Oct2 expression. Data are expressed as the mean ± S.D. values and the experiments were repeated independently at least three times with similar results. *P < 0.05; **P < 0.01 *versus* control group. #*P* < 0.05; ##*P* < 0.01 *versus* T‐treated group.

**Figure 2:**
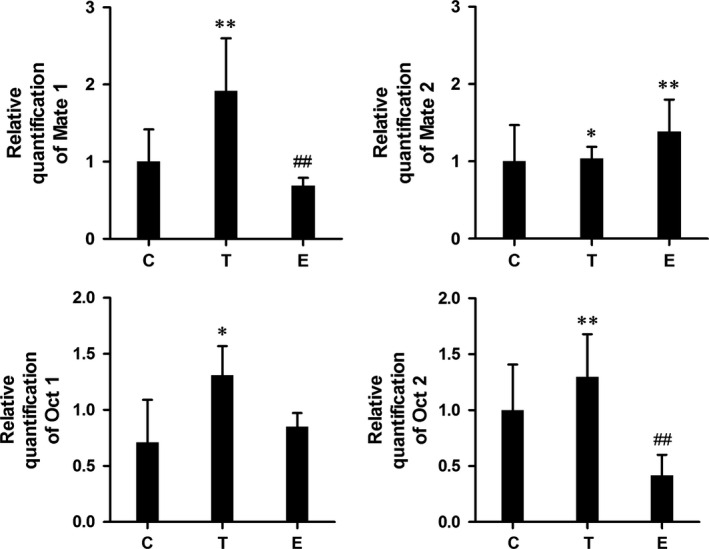
Series 1‐7 days gonadal hormone treatment changed Mate1, Mate2, Oct1 and Oct2 mRNA level among control, testosterone‐treated and oestrogen‐treated groups by quantitative reverse transcriptase PCR. *P < 0.05; **P < 0.01 *versus* control group. #*P* < 0.05; ##*P* < 0.01 *versus* T‐treated group.

Table 1 showed the clinical characteristics of db/db mice before and after gonadal hormone injection in the first series of experiments. There were no significant difference in bw and fasting blood glucose between control and hormone‐treated mice. The 7‐day gonadal hormone injection successfully altered the testosterone and estradiol concentration of mice in both hormone‐treated groups. As expected, serum testosterone concentration was significantly increased in the T‐treated group compared to the control group. In contrast to the control group, the E‐treated group exhibited high serum estradiol level (all *P* < 0.05). Figure 1 showed Mate1, Mate2, Oct and Oct2 protein expression level in kidney tissues in T‐treated group, E‐treated group and control group. There were differences in Mate1 and Oct2 proteins expression levels among three groups (both *P* < 0.01). Unexpectedly, kidney tissue Mate1 protein expression was significantly increased by 100% in the T‐treated group relative to the control group, while reduced by 25% in the E‐treated group (both *P* < 0.05). Similar results also presented in Oct2 protein expression. In contrast to control group, Oct2 protein expression was significantly increased by 100% in the T‐treated mice and reduced by 50% in E‐treated mice (both *P* < 0.05). Moreover, the relative quantification of Mate1 and Oct2 mRNA were significantly increased in T‐treated mice and reduced in E‐treated mice compared to control group (all *P* < 0.01, Fig. 2).

**Table 1:** Clinical characteristics of db/db mice before and after 7‐day gonadal hormone injection in the first series

	Baseline	Control	T‐treated	E‐treated	*P*
Body weight (g)	50.6 ± 0.4	51.7 ± 0.5	51.3 ± 0.7	51.5 ± 0.5	NS
Fasting blood glucose (mmol/l)	18.2 ± 1.1	18.2 ± 1.4	17.9 ± 1.0	18.9 ± 1.3	NS
Testosterone concentration (ng/ml)	1.2 ± 0.02	1.6 ± 0.10	92 ± 23* ^,^ †	0.58 ± 0.08* ^,^ †	0.000
Oestrogen concentration (ng/ml)	0.02 ± 0.01	0.07 ± 0.39	0.38 ± 0.12* ^,^ †	5.00 ± 0.94* ^,^ †	0.000

*P < 0.05 *versus* control group. ^†^ *P* < 0.05 *versus* baseline.

### The effect of gonadal hormone combined with metformin on proteins expression and metformin pharmacokinetics

**Figure 3:**
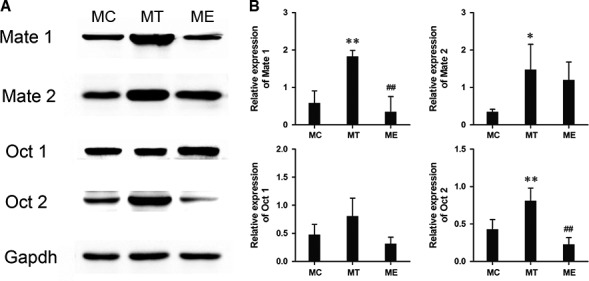
Series 2‐After metformin and gonadal hormone combination treatment, the comparison of Mate1, Mate2, Oct1 and Oct2 proteins expression in kidney tissue among control, MT and ME groups by Western blot. (**A**) Representative Western blot; (**B**) the cumulative results of Western blot analysis of Mate1, Mate2, Oct1 and Oct2 expression. Data are expressed as the mean ± S.D. values and the experiments were repeated independently at least three times with similar results. *P < 0.05; **P < 0.01 *versus* control group. #*P* < 0.05; ##*P* < 0.01 *versus* MT group.

**Figure 4:**
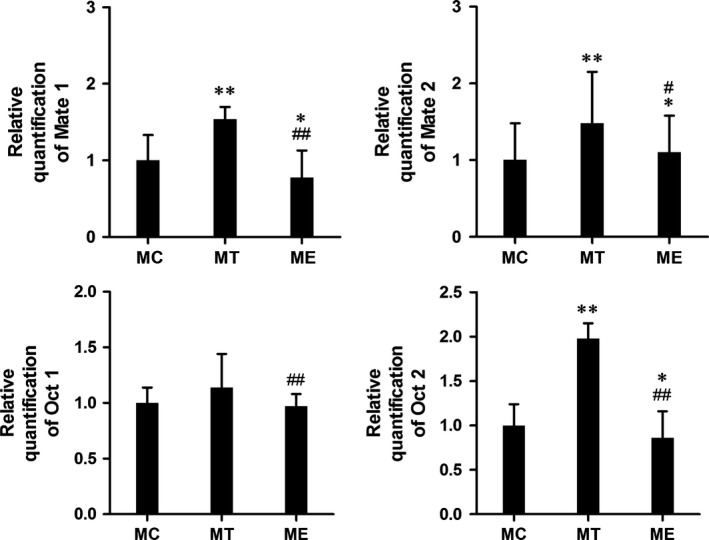
Series 2‐After metformin and gonadal hormone combination treatment. Relative quantification of Mate1, Mate2, Oct1 and Oct2 mRNA among control, MT and ME groups by quantitative reverse transcriptase PCR. *P < 0.05; **P < 0.01 *versus* control group. #*P* < 0.05; ##*P* < 0.01 *versus* MT group.

After combined with metformin, gonadal hormone treatment also changed the protein expression level of Mate1, Mate2, Oct1 and Oct2 in mice kidney. In contrast of control group, Mate1 and Oct2 protein expression was significantly elevated by over 100% in the T‐treated group and reduced by nearly 50% in the E‐treated group (both *P* < 0.01, Fig. 3), which was showing a good agreement with the change in mRNA level (all *P* < 0.05, Fig. 4).

**Figure 5:**
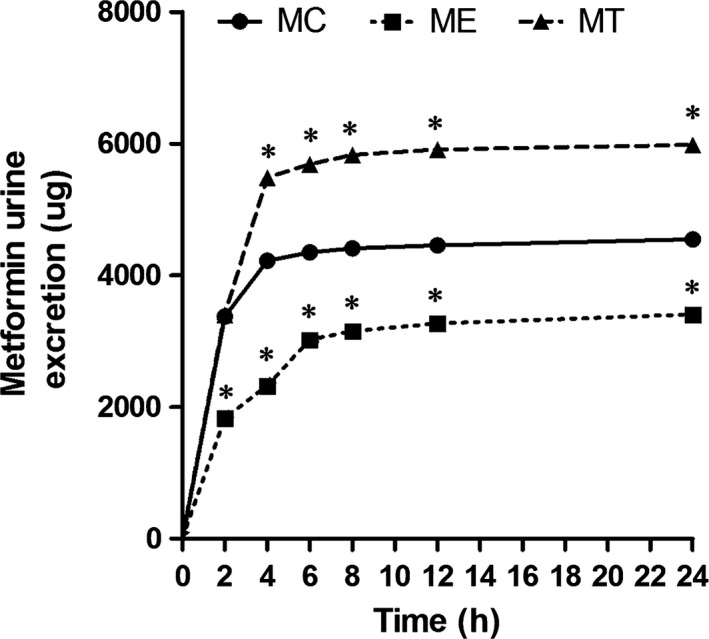
Mean cumulative urinary excretion–time curves of metformin in three mice groups. Intraperitoneal injection of metformin (150 mg/kg) was administered to mice in control group (circles), or MT group (triangles) and ME group (squares) (*n* = 10 each group). *P < 0.05 compared to MC group.

Table 2 showed the metformin concentration in mice plasma and kidney. There were significant differences in plasma and kidney metformin concentration among three mice groups (both *P* < 0.05). Compared to the MC group, mice in MT group had significant lower metformin concentration in plasma and higher metformin concentration in kidney, which were just adverse to mice in ME group (all *P* < 0.05). The metformin cumulative urinary excretion at 2, 4, 6, 8, 12 and 24 hrs was significantly different among three mice groups (all *P* < 0.05, Fig. 5). The total urinary excretion of metformin in MT group was nearly the twice of that in ME group. In contrast of MC group, testosterone strengthened and estradiol retarded the metformin urinary excretion (all *P* < 0.05).

**Table 2:** Metformin concentration among MC, MT and ME groups after 7‐day metformin and gonadal hormone combination treatment

	MC	MT	ME	*P*
Plasma (ng/ml)	293.92 ± 83.27	261.46 ± 79.45*	677.56 ± 72.49** ^,^ ††	0.00
Kidney (ng/ml)	2423.68 ± 794.50	7232.72 ± 176.17**	2128.02 ± 221.69* ^,^ ††	0.04

*P < 0.05; **P < 0.01 *versus* MC group. ^†^ *P* < 0.05; ^††^ *P* < 0.01 *versus* MT group. MC, metformin‐control; MT, metformin‐testosterone; ME, metformin‐oestrogen.

### Correlation analysis between gonadal hormone and protein expression with metformin pharmacokinetics

Spearman's correlation analysis showed that gonadal hormone level was closely associated with Mate1 protein expression (*r* = −0.801), Mate2 protein expression (*r* = −0.650), Oct2 protein expression (*r* = −0.275), Mate1 mRNA (*r* = −0.408), Mate2 mRNA (*r* = −0.603), Oct2 mRNA (*r* = −0.189) and renal metformin concentration (*r* = −0.389; Table 3, all *P* < 0.05).

**Table 3:** Spearman's correlation analysis between sex hormone and other parameters

	Group	Plasma metformin concentration	Renal metformin concentration	Total urine excretion	OCT2 mRNA	OCT1 mRNA	MATE2 mRNA	MATE1 mRNA	OCT2 protein expression	OCT1 protein expression	MATE2 protein expression
MATE1 protein expression	−0.801†	−0.147	0.020	0.569†	0.582†	0.502†	0.508	0.130	0.890†	0.824†	0.248
MATE2 protein expression	−0.650†	−0.569†	0.011	0.068	0.213	0.161	0.240†	0.332	0.310*	0.248	
OCT1 protein expression	−0.366	−0.182	0.191	0.380*	0.543†	0.508†	0.442†	0.090	0.754†	–	
OCT2 protein expression	−0.275*	0.055	0.152	0.451†	0.538†	0.449†	0.376	0.086	–		
MATE1 mRNA	−0.408†	−0.604†	0.174	0.007	0.103	0.502†	0.046	–			
MATE2 mRNA	−0.603†	−0.156	0.556†	0.556	0.002	0.266	–	–			
OCT1 mRNA	−0.560	−0.526†	0.024	0.064	0.711†	–	–	–			
OCT2 mRNA	−0.189†	0.270	0.116	0.231†	–	–	–	–			
Total urine excretion	−0.224	−0.073	0.156	–	–	–	–	–			
Renal metformin concentration	0.389*	0.730	–	–	–	–	–	–			
Plasma metformin concentration	0.519†	–	–	–	–	–	–	–			

*P < 0.05; ^†^ *P* < 0.01.

## Discussion

Metformin is the most frequently prescribed glucose‐lowering medicine worldwide. In this study, we examined the effect of exogenous gonadal hormone on Mates and Octs proteins expression in kidney and metformin pharmacokinetics in db/db mice. To our knowledge, this was the first study which assessed the effect of testosterone and oestrogen on regulating Mate1, Mate2, Oct1 and Oct2 proteins expression in the kidney and urinary excretion of metformin in diabetic mice.

The process of metformin absorption, transport and excretion was associated with many kinds of membrane transporters, manly including Octs and Mates. Encoded by the SLC22A1 and SLC22A2 genes, respectively, Oct1 and Oct2 were members of the solute carrier (SLC) 22 family and expressed separately in the basolateral membrane of hepatocytes and the renal epithelium 16, 17. The uptake of metformin in the hepatocytes by Oct1 and the transportation of metformin to tubular secretion by Oct2 were essential steps for the glucose‐lowering effect 18, 19. Mate1, manly localized at the apical membranes of the renal tubules and bile canaliculi, was considered to be a polyspecific antiporter that directly transported organic cations (OCs) into the urine and bile. Another transporter, MATE2‐K, encoded bySLC47A2, was located in the brush border of the renal epithelium and might also be engaged in metformin excretion 20. The effect of polymorphisms in OCTs and MATEs on metformin disposition and response were discussed in both diabetes and healthy volunteers 21, 22. Our previous studies suggested that the polymorphisms in SLC22A2 (808G→T) and SLC47A1 (rs2289669G→A) could both enhance the long‐term glucose‐lowering effect of metformin *via* influencing its pharmacokinetics in Chinese type 2 diabetes patients 9, 10. With pyrimethamine, the inhibition of the MATE transporter, metformin's area under the concentration–time curve from 0 to 12 hrs was 2.58‐fold greater than control group in healthy Korean male subjects 23. Furthermore, Tsuda *et al*. compared pharmacokinetic characteristics of metformin between wild‐type [Mate1 (+/+)] and Mate1 knockout [Mate1 (−/−)] mice and found that the area under the blood concentration‐time curve of metformin in Mate1 (−/−) mice showed a twofold increase and the renal and secretory clearances of metformin in Mate1 (−/−) mice were approximately 18% and 14% of those in Mate1 (−/−) mice respectively 24.

The association of gonadal hormone with metformin pharmacokinetics attracted our attention for the first time because our previous study found a gender difference in blood lactate levels, which decreased with the increase in age of women. The plasma lactate levels in postmenopausal women were significantly lower than those in premenopausal women 25. We further investigated the effect of gonadal hormone levels on plasma lactic acid levels in type 2 diabetes with and without metformin therapy 11. The result indicated that the lactic acid concentrations increased with the elevation of E_2_ levels but decreased with the increase in T levels in patients with oral metformin 11. Moreover, for eliminating the associated effect of polymorphisms in OCTs on metformin response, we compared the plasma lactate concentrations in patients with the same SLC22A2 808G/T genotype and found the gender difference still existed in the metformin‐treated group 26. Those patients recruited in this study had normal liver and renal function. And there were no significant difference in Cr, BUN and ALT levels between male and female. Therefore, the gender differences in lactate levels are more likely related to the fundamental difference in gonadal hormone levels, not abnormal liver production or renal excretion 26.

Actually, early study had demonstrated that the gonadal steroid hormones were implicated in the regulation of renal OCs transport, which include endogenous compounds (choline, dopamine), drugs (metformin, cimetidine) and xenobiotics tetraethylammonium (TEA, 1‐methyl‐4‐phenylpyridinium) 27. Tetraethylammonium uptake into renal cortical slices and basolateral membrane vesicles of renal proximaltubular cells was higher in male than that of female rat 27. Further study indicated that the gender difference in the expression of rat Oct2 and Mate1 in the kidney was responsible for the gender differences in OC transport activity of the basolateral membrane of renal tubular cells 28, 29, 30, which was consistent with our results in this study. Moreover, treatment of male and female rats with testosterone significantly stimulated the TEA accumulation by renal slices, whereas estradiol treatment caused a decrease in the TEA accumulation by slices from male but not female rats 13.The Oct2 expression was also reported to decrease by the exogenous administration of testosterone in chronic renal failure rats and Madin–Darby canine kidney cells 31, 32. Recently, two androgen response elements in the rat OCT2 promoter region, located at approximately j3000 and j1300, respectively, were suggested to play an important role in the induction by testosterone 14. The role of oestrogen in renal handling of OC *in vivo* and *in vitro* was also fully discussed, claiming that oestrogen status could suppress renal clearance of OCs in mice 33.

Some limitations of this study should also be considered. Firstly, the effect of gonadal hormone on liver protein expression was not clear. According to our previous studies, the effect of MATE1 and OCT2 on metformin elimination by urine was much more important in type 2 diabetes patients. So we focused on the kidney protein expression and urinary excretion of metformin in db/db mice. Besides, considering the little blood volume of db/db mice, this study did not contain the plasma pharmacokinetics analyses of metformin. Last but not least, our study only included male mice, in which the protein expression and metformin excretion in female mice needed to be further analysed.

In summary, this is the first study which compared the bilateral effect of exogenous testosterone and oestrogen on both renal transport proteins expression and metformin pharmacokinetics in db/db mice. We found that testosterone up‐regulated and oestrogen down‐regulate Octs and Mates expression in the kidney of mice, which finally affects urinary excretion and serum concentration of metformin. Considering the high‐frequency usage of metformin in patients with type 2 diabetes and the inter‐individual variability in metformin clinical efficacy, it is of great significance to take the patients’ gonadal hormone level into consideration when assessing the susceptibility of an individual to metformin in order ultimately to achieve the goal of personalized drug therapy.

## Conflict of interest

The authors confirm that there are no conflicts of interest.

## Author contribution

FL and WJ participated in designing the study and revised the manuscript. LA and DZ conducted the mice experiments. LLW detected the metformin concentration. TZ, JY, HL, JXL and FL helped the sample preparation and blood glucose determination. RH analysed the data and wrote the manuscript.
